# A possible direct exposure of the Earth to the cold dense interstellar medium 2–3 Myr ago

**DOI:** 10.1038/s41550-024-02279-8

**Published:** 2024-06-10

**Authors:** Merav Opher, Abraham Loeb, J. E. G. Peek

**Affiliations:** 1 Radcliffe Institute for Advanced Study at Harvard University, Cambridge, MA USA; 2 Astronomy Department, Boston University, Boston, MA USA; 3 Astronomy Department, Harvard University, Cambridge, MA USA; 4 Space Telescope Science Institute, Baltimore, MD USA; 5 Department of Physics and Astronomy, Johns Hopkins University, Baltimore, MD USA

**Keywords:** Solar physics, Solar physics

## Abstract

Cold, dense clouds in the interstellar medium of our Galaxy are 4–5 orders of magnitude denser than their diffuse counterparts. Our Solar System has most likely encountered at least one of these dense clouds during its lifetime. However, evidence for such an encounter has not been studied in detail yet. Here we derive the velocity field of the Local Ribbon of Cold Clouds (LRCC) by modelling the 21 cm data from the HI4PI survey, finding that the Solar System may have passed through the LRCC in the constellation Lynx 2–3 million years ago. Using a state-of-the-art simulation of the heliosphere, we show that during the passage, the heliosphere shrinks to a scale of 0.22 au, smaller than the Earth’s orbit around the Sun. This would have put the Earth in direct contact with the dense interstellar medium for a period of time and exposed it to a neutral hydrogen density above 3,000 cm^−3^. Such a scenario agrees with geological evidence from ^60^Fe and ^244^Pu isotopes. The encounter and related increased radiation from Galactic cosmic rays might have had a substantial impact on the Earth’s system and climate.

## Abstract (web summary)

Modelling suggests that the Solar System may have passed through a cold dense cloud 2–3 Myr ago, in agreement with geological evidence from ^60^Fe and ^244^Pu isotopes, putting Earth in direct contact with the dense interstellar medium with potentially substantial impacts on its climate.

## Main

Most stars generate winds and move through the interstellar medium (ISM) that surrounds them. This motion creates a cocoon (astrosphere) that protects planets from the ISM. The Sun’s cocoon is the heliosphere. The Solar System has been inside the Local Bubble for at least the last 3 Myr, and possibly 10 Myr (ref. ^1^). The conditions near the Sun are not homogeneous, and several partially ionized clouds exist^2^. It is clear that the Solar System has traversed different regions of the local ISM during the past several million years, which has affected its heliosphere. Presently, the Solar System is traversing a local interstellar cloud (LIC) with a relative speed of 25 km s^−1^. The Solar System will be leaving the LIC in the next few thousands of years because of its proximity to its edge^3^.

Here we show that in the ISM that the Sun has traversed for the last couple of million years, there are cold, compact clouds that could have drastically affected the heliosphere. We explore a scenario whereby the Solar System went through a cold gas cloud a few million years ago. Very few works have investigated such encounters with massive clouds^4,5^, in part because the dense ISM needed to sweep away the heliosphere is quite rare. The volume-filling fraction of the dense ISM is less than one part in 1,000. Further, the Sun exists within a large evacuated hot bubble, which has almost no dense gas in it at all; in the vast majority of directions, there are no dense clouds for at least 100 pc in distance from the Sun. The ISM experienced today by the heliosphere is a warm, partially ionized medium with a hydrogen number density of *n*_H_ ≈ 0.2 cm^−3^ and a temperature of *T* ≈ 8,000 K (ref. ^3^). These clouds are plentiful around the Sun, but have too low a density to contract the heliosphere to distances <130 au. The ISM in the vicinity of the Solar System also harbours a few, rare, dense, cold clouds that are called the Local Ribbon of Cold Clouds (LRCC)^6^. The Local Leo Cold Cloud (LLCC)^7^ is among the largest and most studied of the group, and its properties are estimated to be *n*_H_ ≈ 3,000 cm^−3^ and *T* = 20 K (ref. ^8^). Detailed properties have not been recovered for the rest of the LRCC, but they are thought to be qualitatively similar, given the very similar thermodynamic state inferred from 21 cm spectroscopy. The LLCC distance is bracketed to be 11–45 pc (ref. ^9^), and the rest of the LRCC is expected to reside at similar distances.

## Results

### Local Lynx of Cold Clouds

**Fig. 1:**
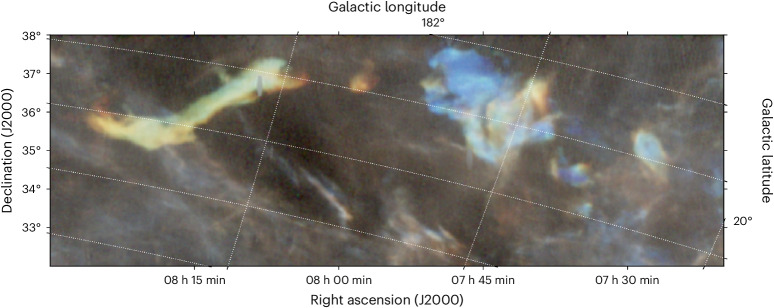
Zoom-in of the LxCCs as seen in 21 cm data from the GALFA-HI survey. In this visualization, three 21 cm velocity channels, each 0.786 km s^−1^ wide, are mapped to red, blue and green. Red represents 8 km s^−1^, green 8.7 km s^−1^ and blue 9.5 km s^−1^, all in the local standard of rest (LSR) frame. The scale is logarithmic from 2 to 40 K brightness temperature. The visualization technique is designed to make the cold clouds stand out in colour (green and red for the left component and iridescent blue for the right component) by taking advantage of the narrowness of their velocity profiles compared to the warmer background gas much farther away. GALFA-HI survey data from ref. ^67^.

**Fig. 2:**
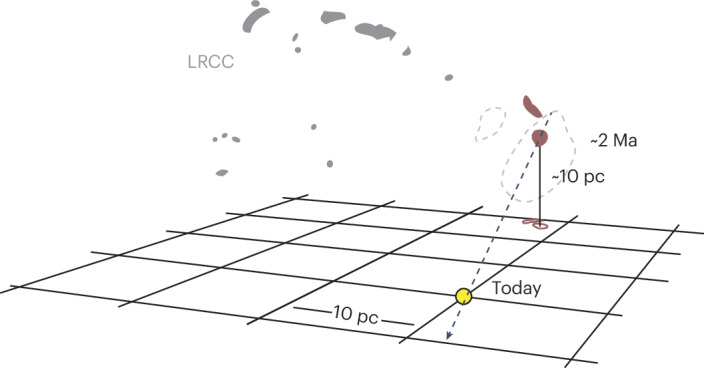
Cartoon of the LRCC and its three-dimensional velocity. We show the LRCC with two LxCCs highlighted in red. The Sun is shown in the Galactic plane at (0,0), with a 10 pc grid for scale. The black dashed line represents the path of the Sun in the LRCC rest frame. The grey dashed lines represent the 68% confidence interval of the past path of the Sun, which contains only 1.3% of the total sky.

**Fig. 3:**
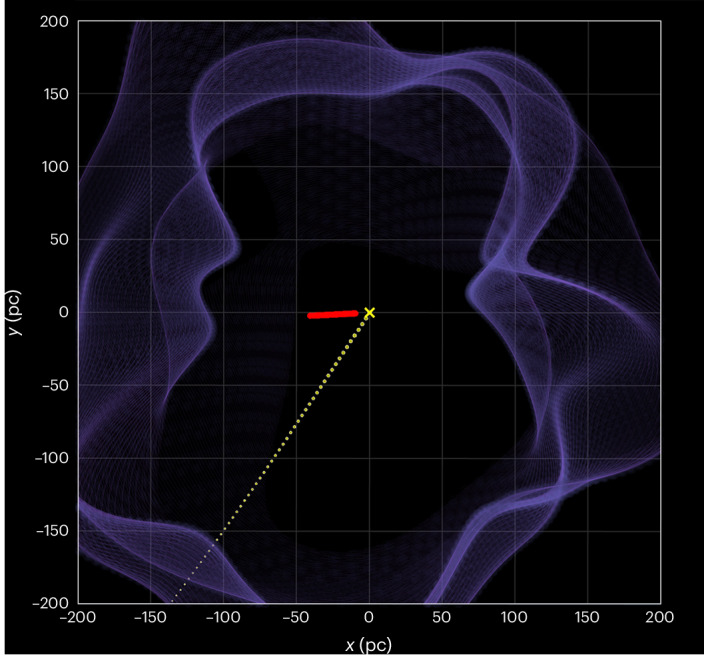
The collision between the LxCCs and the Sun is shown in the LSR frame using an interactive graphic. For this plot, 100 draws made from the velocity and distance distributions were tracked backwards in time. Each red point represents the path of the LxCCs from 5.75 Ma to the present. The dotted yellow line is the velocity field of the Sun. The blue surface represents the edge of the Local Bubble^64^ (Supplementary Video 1). Credit: Catherine Zucker (https://faun.rc.fas.harvard.edu/czucker/Paper_Figures/Interactive_LxCC.html).

We show below that the distance and velocity characteristics of the LRCC are such that there is at least a 1.3% chance that the heliosphere encountered the tail of the LRCC 2 million years ago (Ma) in the direction of the Lynx constellation. We name that portion the Local Lynx of Cold Clouds (LxCCs). The LxCCs represent nearly half of all the mass of the LRCC and are more massive than the more well-studied LLCC, presuming an identical distance. The LRCC has a very placid and smooth velocity field, and it is a thin band that stretches across nearly 90° of the sky. Taking advantage of this remarkably well-organized velocity structure, Haud^6^ modelled it as a subarc of a rotating, expanding, moving ring in space with five parameters. Here we propose a more modest, three-parameter model, in which the LRCC simply moves as a fixed, non-rotating structure, and solve for the full three-space motion of the cloud. We use 21 cm data from the all-sky HI4PI survey (HI4PI Collaboration^10^), from which we isolate cold structures and fit with narrow-line Gaussians. We recover spatial and velocity structures consistent with the results Haud^6^ recovered from a lower-resolution dataset. We fit the velocity field as a fixed, non-rotating structure. The relative velocity in Galactic coordinates between the LLCC and the Sun is (∆*U*, ∆*V*, ∆*W*) = (−13.58, −1.40, 3.70) km s^−1^ or a velocity of 14.1 km s^−1^ towards *l* = 186° and *b* = 15° (*l* is Galactic longitude and *b* is Galactic latitude). We found that the 1*σ* error region of the direction of flow of these clouds covers 1.3% of the sky (576 square degrees), including the tail of the LRCC itself (Figs. 1 and 2). This probability could be larger because the LRCC is a wide structure that spans a large portion of the sky. As shown in Fig. 3, in our Monte Carlo simulation, the LRCC as a whole is wildly unstable in the ISM, and thus, it was probably larger in the past. So, the low chance of collision is a lower limit.

As the clouds have a positive (outgoing) velocity, the coincidence of the cloud on the sky within the cloud velocity error circle indicates that the Sun crossing the clouds is consistent with the model. The distance to the LLCC, the largest cloud in the LRCC, is known to be between 11 and 45 pc, which allows us to compute a 68.3% confidence interval for the LxCCs of 22 to 59 pc (‘LLRC detection, distance and velocity statistics’ in Methods). We found the 68.3% confidence interval of the radial velocity to be 11.4–15.6 km s^−1^. These parameters translate to the Sun crossing the position of the clouds between 1.57 and 4.2 Ma. It, therefore, appears compelling that the Solar System passed through a cold, dense, ISM cloud 2 Ma (Fig. 3). Note that these clouds are anomalous and unexplained structures in the ISM, and their origin and physics are not well understood^8^. We have assumed here that these clouds have not undergone any substantial change over the last 2 Myr, though future work may provide more insight into their evolution.

### Heliosphere 2 Ma

We simulated the interaction of the heliosphere 2 Ma with the LxCCs. The distance to the edge of the heliosphere is currently ~130 au, as measured by Voyagers 1 and 2 (ref. ^11^). As our simulation demonstrates, the momentum deposition by the large hydrogen density of the cloud shrinks the heliosphere to a scale that is much smaller than the Earth’s orbit around the Sun and brings the Earth and the Moon in direct contact with the cold ISM. Such an event may have had a dramatic impact on the Earth’s climate.

Our computational code considers a single ionized component and four neutral components^12^, although for this run, we used only the ISM component, which is orders of magnitude more abundant than the heliosheath and supersonic solar-wind components. We used inner boundary conditions for solar-wind conditions at 0.1 au (or 21.5 solar radii). The parameters adopted for the solar wind were based on the well-benchmarked Alfven-driven solar-wind solution^13^. The grid was highly resolved at 1.07 × 10^−3^ au near 0.1 au and 4.6 × 10^−3^ au in the region of interest, including the tail (Supplementary Fig. 1). The run was performed for 44 years (see ‘Description of the numerical model’ in Methods for a description of the coordinate system, grid and model details). For the ISM outside the heliosphere, we adopted the characteristics of the LLCC^8^, namely, *n*_H_ = 3,000 cm^−3^ and *T* = 20 K. We included a negligible ionized component (*n*_i_ = 0.01 cm^−3^ and *T* = 1 K) and ignored the interstellar magnetic field as its pressure is negligible compared to the ram pressure of the cold cloud. We adopted the relative speed between the Sun and LxCCs $(\Delta U,\Delta V,\Delta W\;)=\left(-13.58,-\mathrm{1.40,3.70}\right)$ km s^−1^ or a velocity of 14.1 km s^−1^. We rotated the system so that the flow is in the *z–x* plane with the ISM approaching from the -x direction. The neutral H from the cold cloud impinged on the heliosphere with speeds of *U*_*x*_ = 14.1 km s^−1^, *U*_*y*_ = 0 km s^−1^ and *U*_*z*_ = 1.1 km s^−1^ (see ‘ISM conditions’ in Methods for details).

The numerical model includes charge exchange between the neutrals and ions^12^, as well as the Sun’s gravity, which plays an important role in focusing the gas flow. Neutral H atoms are included through a multi-fluid description that is appropriate for the high densities^14,15^. Two fluids are used, one between the pristine ISM and the bow shock that forms ahead of the heliosphere and one that captures the heated and decelerated population between the bow shock and the heliopause (HP). We neglected radiation pressure from the Lyα line of hydrogen atoms since these cold dense clouds are optically thick to Lyα photons^4^. We neglected photoionization, as its contribution is an order of magnitude smaller than that of charge exchange at these distances (see ‘Description of the numerical model’ in Methods for details).

**Fig. 4:**
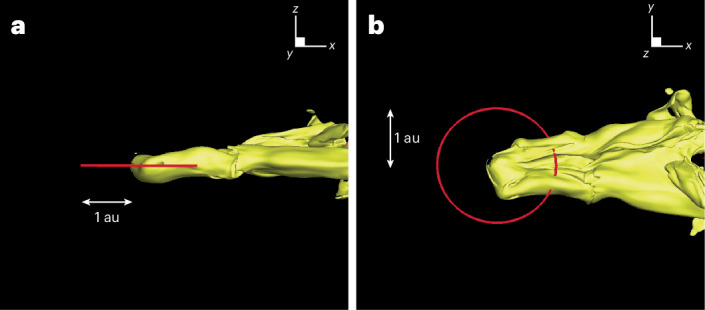
Three-dimensional image of the heliosphere. **a**,**b**, Side view in (*x*,*z*) coordinates (**a**) and top view in (*x*,*y*) coordinates (**b**) (‘Description of the numerical model’ in Methods). The orbit of Earth around the Sun is plotted in red. The isosurface of the heliosphere is plotted with speed of 100 km s^−1^. We plotted the tail out to 4 au.

**Fig. 5:**
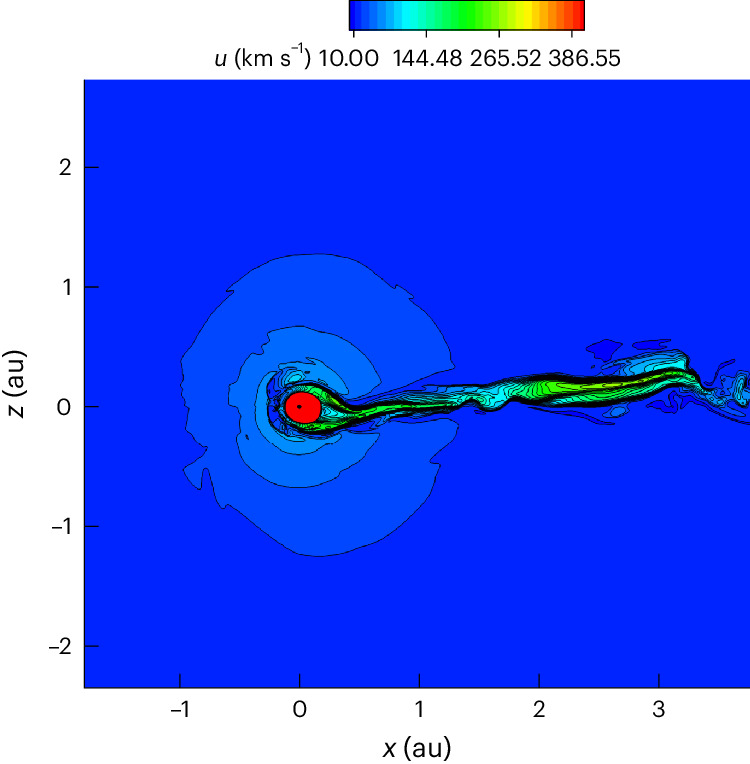
View of the heliosphere 2 Ma. The heliosphere at the end of the simulation at 44 years in the meridional plane at *y* = 0 au (for the model coordinate system, see ‘Description of the numerical model’ in Methods). Contours are speed. The heliosphere shrinks to 0.22 au at the nose. It maintains a long cometary shape and exposes all the planets to the cold dense ISM material.

**Fig. 6:**
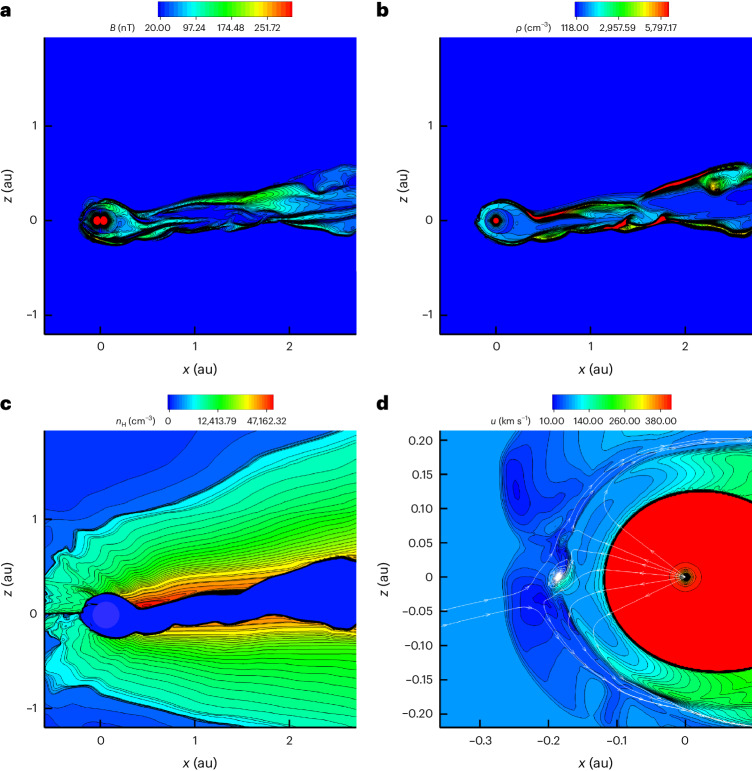
A closer view of the heliosphere 2 Ma. This figure provides a closer view of Fig. 5. Panels are shown at the end of simulation at 44 years in the meridional plane at *y* = 0. The coordinate system is such that the *z* axis is parallel to the solar rotation axis, the *x* axis is oriented in the direction of the interstellar flow (which points 5° upward in the *x*–*z* plane) and the *y* axis completes the right-handed coordinate system where the Sun is at rest at the centre. **a**, Magnetic field. **b**, Ion density. **c**, Neutral density. **d**, Speed.

Figures 4 and 5 show the heliosphere as a result of the interaction with LxCCs 2 Ma. The heliosphere shrinks to 0.22 ± 0.01 au, which is well within the Earth’s orbit, thus exposing the Earth (and all the other Solar System planets for most of their trajectories) to the ISM, which has neutral densities of 3,000 cm^−3^ (Fig. 6c). Due to gravity, the neutral density increases as the cold cloud encounters the heliosphere, so that inner planets, such as Mercury and Venus (at distances of 0.39 and 0.72 au), will encounter densities of 7,000 cm^−3^. The size of the heliosphere can be compared with the stand-off distance expected from analytic estimations. One can estimate analytically the stand-off distance^16^ as approximately ${r}_\mathrm{E}\sqrt{{\rho}_\mathrm{E}{v}_\mathrm{E}^{2}/{\rho}_{\infty }{v}_{\infty }^{2}}$, where *r*_E_, *v*_E_ and *ρ*_E_ are the radius, speed and density of the solar wind at Earth and *ρ*_∞_ and *v*_*∞*_ are the density and speed at infinity of the ISM. Taking the values *ρ*_E_ = 5.71 cm^−3^, *v*_E_ = 417 km s^−1^, *ρ*_*∞*_ = 3,000 cm^−3^ and *v*_*∞*_ = 14.1 km s^−1^, the stand-off distance is 1.3 au. The neutral density due to gravity increases to 10,000 cm^−3^ ahead of the heliosphere and the neutral speed to 50 km s^−1^, making the same estimate 0.2 au, which agrees very well with the simulation results.

The supersonic solar wind goes through a termination shock (TS; Fig. 5) before reaching equilibrium with the cold cloud. The heliosphere has a cometary shape with a long tail (Fig. 5) that is unstable. Due to their short mean free path (~0.01–0.1 au), the neutrals are quickly depleted across the HP (Fig. 6c), setting a strong gradient for the ram pressure. The heliosphere reaches equilibrium with the cold cloud at the HP between the compressed solar magnetic field and the ram pressure of neutrals ahead of the HP (Supplementary Fig. 2). Gravity increases the density from 3,000 cm^−3^ and the speed from 14.1 km s^−1^ of neutrals at large distances to 10,000 cm^−3^ and to 50 km s^−1^, respectively, near the HP (Fig. 6c,d).

A bow shock is formed in the ISM (Fig. 6d). The density of neutrals increases to ~10,000 cm^−3^ near the bow shock. In that region, the temperature also increases to ~10^5^ K. The cooling time *τ*_cooling_ for different densities and temperatures^17^ varies in the range log_10_[*τ*_cooling_ (s)] ≈ 10–15. The lower limit on the cooling time is ~300 years, which is much longer than the dynamic time to form the shock *τ*_dyn_. For the relative speed with which the Earth moves through the ISM, namely, 27 km s^−1^ ≈ 6 au yr^−1^, and a bow shock thickness of about less than 1 au, it follows that *τ*_dyn_ ≈ 1 year, which is much shorter than *τ*_cooling_. Hence, radiative losses can be neglected.

The heliosphere 2 Ma was very different from the heliosphere of today^12^. There was no hydrogen wall as the number of ions ahead of the heliosphere was negligible. The heliosphere was so close to the Sun that the solar magnetic field was radial (Fig. 6a) and the heliosheath plasma confinement (Fig. 6b) did not take place^12^. The flow in the heliosheath was fast (~110–260 km s^−1^) (Fig. 5), and the ram pressure was larger than the magnetic pressure. The Rayleigh–Taylor-like instability that currently occurs in the heliosheath^18^ and drives the current heliosphere to have a short tail was absent. Because of the short mean free path, there were almost no neutrals inside the heliosheath, and the density gradient in the heliosheath was absent as well. The TS shifted to distances as close as 0.12 au from the Sun. The present-day TS is weakened by pickup ions compared to the much stronger compression ratio of 3.7 of the TS during the passage of the cold cloud. This has consequences for accelerating particles to high energies. We expect that the stronger shock accelerated particles more efficiently than the current TS, which is mediated by pickup ions^19^. Future work is needed to explore the resulting non-thermal emission and its consequences for planets around the Sun and other stars.

Figure 5 shows the elongated, high-speed tail of the ancient heliosphere. Such elongated tails may be common for solar-mass stars just born in dense interstellar environments, like molecular clouds, and they may have been misinterpreted in the past as jets^20^.

### ^60^Fe and ^244^Pu isotopes

By studying geological radioisotopes on Earth, we can learn about the past of the heliosphere. ^60^Fe is predominantly produced in supernova explosions^21^ and becomes trapped in interstellar dust grains. ^60^Fe has a half-life of 2.6 Myr, and ^244^Pu has a half-life of 80.7 Myr. ^60^Fe is not naturally produced on Earth, and so its presence is an indicator of supernova explosions within the last few (~10) million years. ^244^Pu is produced through the r-process that is thought to occur in neutron star mergers^22^. Evidence for the deposition of extraterrestrial ^60^Fe onto Earth has been found in deep-sea sediments and ferromanganese crusts between 1.7 and 3.2 Ma (refs. ^23–27^), in Antarctic snow^28^ and in lunar samples^29^. The abundances were derived from new high-precision accelerator mass spectrometry measurements. The ^244^Pu/^60^Fe influx ratios are similar at ~2 Ma, and there is evidence of a second peak at ~7 Ma (refs. ^23,24^). In addition, cosmic ray data assembled by the Advanced Composition Explorer spacecraft measured the ^60^Fe abundance as well^30^. This study estimated the time required for transport to Earth and concluded that the cosmic rays diffused from a source closer than a distance of thousands of parsecs. Studies have attributed the two peaks in ^60^Fe to supernova explosions within 100 pc over the last 10 Myr that formed the Local Bubble^24^. Processes that brought ^244^Pu to Earth include supernova ejecta.

Other studies suggest that nearby supernova explosions within ~10–20 pc could have produced the above isotopes^31^. In particular, the heliosphere would collapse to distances less than 1 au if a supernova is closer than 10 pc. This scenario requires fine-tuning, as this distance is very close to the so-called kill radius of 8 pc (ref. ^32^), the distance necessary to initiate a mass extinction. A close supernova explosion contradicts the recent model of the Local Bubble formation^33^, which indicates that the Local Bubble originated when supernovae exploded 14 Ma near the centre of the Local Bubble at a distance much larger than 10 pc. For supernovae at further distances (such as the more recent study^34^ that places a supernova at 50 pc), it has yet to be shown that sufficient ^60^Fe can be deposited onto Earth if ^60^Fe is embedded in large interstellar dust grains, although some researchers^35^ have started to investigate this (albeit the complex filtration of the heliosphere and its magnetic field affecting its propagation to Earth has yet to be investigated). In particular, the propagation of dust in a realistic heliospheric magnetic field has yet to be studied. Our proposed scenario agrees with the geological evidence from ^60^Fe and ^244^Pu isotopes that Earth was in direct contact with the ISM during that period.

## Discussion

Previous works have explored the effect of the different environments experienced by the heliosphere as the Sun travels through the ISM on the filtration of Galactic cosmic rays (GCRs) that affect Earth^36–40^. The effect of GCRs on Earth’s atmosphere and climate is still uncertain^41,42^. Some previous works have speculated that encounters of the heliosphere with molecular clouds could affect Earth’s environment^4,5^. Our proposed scenario implies that all planets in the Solar System were exposed simultaneously to the ISM. The scenario does not require the absorption of ^60^Fe and ^244^Pu into dust particles that deliver them specifically to Earth, like the scenario with nearby supernova explosions^24,31^.

There is a need to explore the physical connections between heliospheric compression and planetary climates and atmospheres. The consequences of the Earth being exposed to the ISM are outside the present work. Here we just comment briefly on some of them. First, there is a possible consequence for Earth’s climate. Large amounts of neutral hydrogen as a result of an encounter with cold clouds with densities above 1,000 cm^−3^ will alter the chemistry of Earth’s atmosphere. This needs careful examination including the physics of cloud formation. Very few works have investigated the climatic effects of such encounters quantitatively in the context of encounters with dense giant molecular clouds. Some argue that such high densities would deplete the ozone in the mid-atmosphere (50–100 km) and eventually cool the Earth^43,44^. This work should be revisited with modern atmospheric modelling. This cooling is aligned with what is seen for oxygen isotopes measured in the microscopic skeletons of foraminifera on the sea floor^45^. It has been suggested that climate changes around this time could have affected human evolution^46–49^. The hypothesis is that the emergence of our species *Homo sapiens* was shaped by the need to adapt to climate change^50^. With the shrinkage of the heliosphere, the Earth was exposed directly to the ISM.

Another effect would be increased radiation from an increased flux of GCRs. Voyagers 1 and 2 showed that the heliosphere shields the GCRs for intensities 70 MeV–5 GeV by 80% (refs. ^11,51^). During the passage through a cold cloud, Earth is exposed to the bare GCR intensity, which is enhanced further by the compression of the cold cloud if the GCRs are trapped within the cloud, although some studies^52^ argue that the GCR spectra in dense clouds are like the local ISM. Detailed modelling of GCR diffusion is needed to explore the impact of GCRs on climate and habitability. Additionally, the passage through such a dense cloud would have an additional effect on Earth’s atmosphere through interstellar dust accumulation^53^. This also should be investigated.

Although the coincidence of the Sun’s past motion with these rare clouds is truly remarkable, the turbulent nature of the ISM and the small current angular size of these clouds mean that the past location error ellipse is much larger than the clouds and, absent any other information, the probability of their encounter is measured to be low.

Connecting Gaia-informed three-dimensional dust maps with velocity-resolved spectral-line gas maps in the solar vicinity should provide constraints on both the density and dynamics of the local ISM. In the future, these constraints will shed new light on how often the Sun would have encountered clouds capable of shrinking the heliosphere to sub-astronomical unit scales.

We hope that our present work will incentivize future works detailing the climate effects due to an encounter of the heliosphere with the LRCC and possible consequences for evolution on Earth.

## Methods

### Description of the numerical model

The cold, thermal, solar-wind ions and hot pickup ions are treated as a single species. The neutral hydrogen component is captured with a four-fluid approximation^14,15^, although for this problem, only the supersonic component and the cold pristine ISM take part in the interaction. A bow shock is formed in the ISM.

We neglect radiation pressure from the Lyα line of hydrogen atoms as these cold dense clouds are optically thick to Lyα photons, which have an optical depth *τ* much larger than unity. At the column density of hydrogen atoms in LxCCs, *N* ≈ [10^3^ cm^−3^] × [pc] ≈ 10^21^ cm^−2^. The Lyα cross section at resonance is *σ* ≈ 7 × 10^−11^ cm^2^ (ref. ^54^) and so *τ* ≈ *Nσ* ≈ 10^11^. Hence, radiation was inferred to play a smaller role than gravity (same as in ref. ^4^). This is different than in current ISM conditions, where radiation pressure is comparable to gravity^55^. We neglect photoionization as its contribution is an order of magnitude smaller than that of charge exchange at these distances.

The coordinate system in the computational model is such that the *z* axis is parallel to the solar rotation axis, the *x* axis is oriented in the direction of the interstellar flow (which points 5° upward in the *x*–*z* plane) and the *y* axis completes the right-handed coordinate system in which the Sun is at rest at the centre.

Future work could explore the current scenario with more advanced codes where the cold solar-wind and suprathermal ions are treated as separate components^56^ or the neutral hydrogen atoms are treated kinetically^57^. We do not expect, though, any major changes from the current results. The density of neutrals is so high that a fluid treatment is appropriate^4^. The separation of thermals and suprathermals will enhance our results and bring the heliosphere further in. This is because the pickup ions charge exchange (the mean free path for kilo-electronvolt pickup ions is ~0.01 au for densities as high as 10^3^ cm^−3^) and leave the system, thus deflating the heliosphere.

### Inner boundary

The inner boundary was placed at 0.1 au (or 21.5 solar radii). The parameters adopted for the solar wind at the inner boundary are *v*_SW_ = 417 km s^−1^, *n*_SW_ = 5.71 × 10^2^ cm^−3^ and *T*_SW_ = 2.59 ×10^5^ K based on the Alfven-driven solar-wind solution^13^. The magnetic field is given by the Parker spiral magnetic field^58^ with *B*_SW_ = 1.72 × 10^2^ nT at the equator. We used a monopole configuration for the solar magnetic field (as in refs. ^12,59^). This description, while capturing the topology of the field lines, does not capture the change of polarity with solar cycle or across the heliospheric current sheet. This choice, however, minimizes artificial reconnection effects, especially in the heliospheric current sheet. We assumed that the magnetic axis is aligned with the solar rotation axis.

### ISM conditions

For the ISM outside the heliosphere, we adopted the characteristics of LLCC^8^, namely, *n*_H_ = 3,000 cm^−3^ and *T* = 20 K. We included a negligible ionized component (*n*_i_ = 0.01 cm^−3^ and *T* = 1 K) and ignored the interstellar magnetic field as its pressure is negligible compared to the ram pressure of the cold cloud. Both were streaming with a speed of *U*_*x*_ = 14.1 km s^−1^, *U*_*y*_ = 0 km s^−1^ and *U*_*z*_ = 1.11 km s^−1^ (see ‘Coordinate transformation from Galactic coordinates to model coordinates’).

### Grid resolution

The grid extends ±50 au in *y* and *z* and −20–50 au in *x*. We cover all regions of interest with a high grid resolution. The smallest grid cell is 1.07 × 10^−3^ au near the inner boundary and 4.6 × 10^−3 ^au in the region of interest including the tail (Supplementary Fig. 1). The numerical scheme is a second-order Linde scheme, so error bars are within two grid cells^60^. The TS in the upstream direction is at 0.14 ± 0.002 au. The resolution at the HP is 0.004 au, so the HP is at 0.22 ± 0.008 au. The heliosheath width is then 0.08 ± 0.008 au upstream. The tail direction was resolved with a resolution of ±0.002 au extending to 5 au. In particular, the resolution used (0.22 ± 0.008 au) was more than sufficient to resolve the most important boundary, which is the location of the HP upstream at 1 au.

### LLRC detection, distance and velocity statistics

We followed a procedure for cloud measurement like that used in ref. ^61^ but focused on the LRCC and with updated data. We accessed the 21 cm data radio cubes from the HI4PI survey^10^, which cover the hyperfine line for hydrogen emission in the Galaxy. For the data that correspond to the region of the LRCC, we first performed a Gaussian smoothing in the image domain, with a sigma of 10 arcmin to enhance the signal-to-noise ratio of the faint emission. We then performed unsharp masking (or high-pass filtering) in the velocity domain, subtracting a three-channel (3.86 km s^−1^) boxcar-smoothed data cube. We then selected any position whose brightness temperature (*T*_ISM_) met the criterion

$${T}_{\mathrm{ISM}} > 0.2\,{\rm{K}}/({T}_{\mathrm{data}}/1\,{\rm{K}}+0.5),$$

which successfully selects for narrow, cold features that are neither produced by very high column density regions nor by noise. We then fitted these lines of sight over a narrow velocity window (11.59 km s^−1^) with a Gaussian plus a slope, the latter representing background emission. Any fits that were at velocities inconsistent with the LRCC were discarded as were any emission lines broader than 2.7 km s^−1^, following ref. ^61^. This set of positions and velocities (shown in Fig. 1) comprise our LRCC data and are visually very consistent with what is seen in ref. ^6^. These velocities were translated into the barycentric frame, the velocity frame of the barycentre of the Solar System, from the local standard of rest (LSR) using:

$${V}_{\mathrm{Bary}}={V}_{\mathrm{LSR}}-9\cos(l)\cos(b)-12\sin(l)\cos(b)-7\sin(b),$$

which represents the ‘dynamical’ definition of the LSR from the International Astronomical Union. We then fitted these barycentric velocities under the assumption that the LRCC is moving as a fixed, non-rotating body using a standard least-squares procedure. Although the cloud is quite placid, its residual turbulent structure guarantees that no simple velocity model will ever fully capture the data. As the residuals from the fit are clearly correlated, we modelled the error on our velocity fit by performing block bootstrapping^62^, in which large sections of the cloud were resampled with replacement, which allowed us to more accurately capture the effect of spatially correlated residuals to the fit and does not assume independent and identically distributed data. We blocked regions that correspond to HEALPix tiles^63^ for NSIDE = 4 and used the resulting draws to refit the data and construct our error regions. Note that changing the block size by factors of 4 did not qualitatively change our results. The resulting 1*σ* contours cover 1.3% of the sky and include the LxCCs.

The distance constraints towards the LLCC provide a 100% confidence interval for the distance to LLCC of 11 to 45 pc. From this, we derived the probability distribution function (PDF) of the distances to the clouds of interest, the LxCCs. We believe it is quite reasonable to make the assumption that the LRCC has a common origin; it is the only structure of its kind in the sky, it forms a roughly straight line and it has a remarkably smooth velocity field^6^. The width of the ribbon does not seem to vary significantly, with the envelope of the ribbon consistently between 3° and 5° wide. Using this information, we assumed that the cloud is no more than twice as far away at one end than the other, with the LxCC end of the LRCC 50° on the sky away from the LLCC and the opposite end of the LRCC 40° away from the LLCC. Using this information, we ran a Monte Carlo experiment, randomly selecting a distance for the LLCC from along the 11–45 pc 100% confidence interval, randomly selecting a plane-of-sky angle for a straight LRCC and rejecting any solutions in which one side was more than two times closer than the other. This produced a distribution for the distance to the LxCCs that has a 68% confidence interval that spans from 22 to 59 pc. We used the full distance PDF to compute the range of times of collision.

We calculated the possibility of the Sun having passed this close to a cloud as dense and massive as the LxCCs 2 Mya by chance. There are no other known dense ISM clouds within the Local Bubble, the very low-density volume of the ISM that surrounds the Sun, whose closest wall is 80 pc away^64^. The LxCCs are the most massive of the LRCC clouds and are significantly smaller than our error ellipse, which covers only 1.3% of the sky. This represents the chances that the Sun would pass as close to such a massive cloud at all in its recent history by chance, not approximately 2 Mya.

To compute the probability of a chance passage between the Sun and this densest cloud as consistent with the ^60^Fe event recorded, we considered the PDF of the motion between the Sun and the cloud. The Sun is a member of the old thin stellar disk, which has a radial velocity dispersion of 35 ± 5 km s^−1^ and a vertical velocity distribution of 25 ± 5 km s^−1^ (refs. ^65,66^). The 21 cm gas velocity dispersion in the disk is typically quite a bit smaller (~10 km s^−1^), and thus, the relative velocity PDF of the velocity between a typical Sun-like star and a cloud is dominated by the stellar velocity distribution. If we draw randomly from this velocity distribution and place the cloud within the nearest wall of the Local Bubble at random, the chance of such a close passage of the Sun to such a cloud is 1%.

### Coordinate transformation from Galactic coordinates to model coordinates

The relative velocities between the Sun and the cold cloud in Galactic coordinates are *U*_*x*_ = −12.1, *U*_*y*_ = −12.6 and *U*_*z*_ = 20.9 km s^−1^, which correspond to Galactic coordinates (latitude, longitude) = (570.11°, −133.84°). Converting Galactic to Ecliptic coordinates (https://lambda.gsfc.nasa.gov/toolbox/tb_coordconv.cfm) for the J2000 epoch, in Ecliptic coordinates, this corresponds to (latitude, longitude) = (1.46°, 150.48°).

In the HCI coordinate system (our model), this corresponds to (latitude, longitude) = (−5.54°,75.67°) or to coordinate vector (*x*_HCI_, *y*_HCI_, *z*_HCI_) = (0.2464,0.9644, −0.0965). This corresponds to the relative speeds in HCI of *U*_*x*,HCI_ = 6.71 km s^−1^, *U*_*y*_,_HCI_ = 26.27 km s^−1^ and *U*_*z*_,_HCI_ = −2.63 km s^−1^.

## Supplementary information

Supplementary Information Supplementary Figs. 1 and 2.

Supplementary Video 1 Animation of Fig. 3.

## Data Availability

The data that support the findings of this study are all publicly available.

## Code availability

Our model is the outer heliosphere component of the Space Weather Modeling Framework and is publicly available at http://csem.engin.umich.edu/tools/swmf/.
