# Supplementary material for: A possible direct exposure of the Earth to the cold dense interstellar medium 2–3 Myr ago
Source: Nat Astron. 2024 Jun 10;8(8):983–90. doi: 10.1038/s41550-024-02279-8 (PMC11335566; doi:10.1038/s41550-024-02279-8)
Supplement: Supplementary file 1 — Supplementary Figs. 1 and 2. [file 41550_2024_2279_MOESM1_ESM.pdf]

nature astronomy

Supplementary information

https://doi.org/10.1038/s41550-024-02279-8

A possible direct exposure of the Earth to the cold dense interstellar medium 2–3 Myr ago

In the format provided by the authors and unedited

Supplementary Information

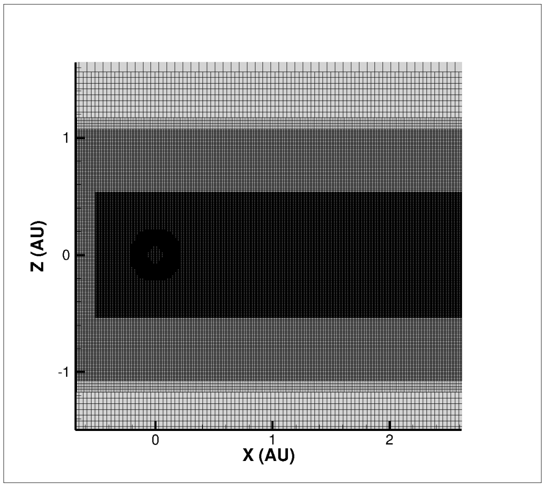

Supplementary - Figure 1. Grid is shown in a zoom region around the region of interest in the meridional plane at y=0.

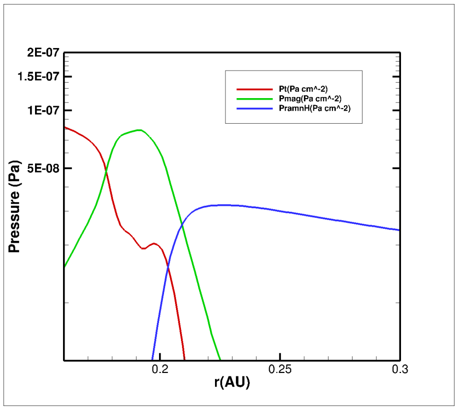

Supplementary - Figure 2. Cut through the nose showing the pressures across the heliopause (located at 0.22 ± 0.01AU) – blue line is the ram neutral pressure; green line the magnetic pressure; the red line the thermal pressure.
